# The hidden dangers lurking at home: Unveiling the prevalence of leftover antibiotics and its associated factors among Lebanese households

**DOI:** 10.1016/j.puhip.2024.100485

**Published:** 2024-03-02

**Authors:** Wadih Saadeh, Stephanie Chaccour, Deema Rahme, Nathalie Lahoud, Nadine Saleh

**Affiliations:** a Faculty of Pharmacy, Lebanese University, Hadat, Lebanon; b Pharmacy Practice Department, Faculty of Pharmacy, Beirut Arab University, Beirut, Lebanon; c INSPECT-LB (Institut National de Santé Publique, d'Épidémiologie Clinique et de Toxicologie-Liban), Beirut, Lebanon; d Faculty of Pharmacy, Lebanese International University, Beirut, Lebanon; e Faculty of Public Health, Lebanese University, Fanar, Lebanon

**Keywords:** Antibiotics, Leftovers, Prevalence, Lebanon, Households, Self-medication

## Abstract

**Background:**

Antimicrobial Resistance (AMR) is a major global concern. Irrational use of antibiotics including self-medication (SM) with leftovers without a medical prescription can be a leading cause. This study aimed to investigate the prevalence and related factors of leftover antibiotics (LA) in Lebanese households.

**Study design:**

A cross-sectional study of the Lebanese population was conducted between March and October 2022.

**Methods:**

Through random proportional stratified sampling, a total of 494 families participated in this study. Data collection was carried out through phone calls using a comprehensive and reviewed questionnaire. The data was then analyzed using SPSS version 26. Logistic regression was utilized to identify the factors associated with LA, with the presence of LA in households as the dependent variable and other factors such as age, region of residence, and presence of elderly individuals at home as the independent variables.

**Results:**

Among selected households, 118 households (23.89%) had LA. The most common type of antibiotic found was penicillin (59.84%). Most of the LA were in the form of tablets and capsules (94%) with valid expiration dates (87%). Antibiotics were mainly prescribed by doctors (61%), and the main reason for prescribing was acute respiratory tract infections (47.46%). SM was reported by 42.37% of the families with LA. A family with elderly patients (p = 0.002; OR = 2.23; 95% CI = 1.33–3.73) and those residing in Mount Lebanon (p = 0.019; OR = 2.28; 95% CI = 1.14–4.56) had significantly higher odds of having LA.

**Conclusion:**

Leftover antibiotics were found in nearly a quarter of the addressed Lebanese families. Therefore, public educational campaigns should be launched to limit injudicious antibiotic use including SM, and to promote proper disposal of any leftovers. It is also crucial to adopt the One Health approach by developing national programs for the safe disposal of LA and implementing regulations to restrict the distribution of antibiotics in pharmacies without a prescription.

**Advances in knowledge**

LA are a critical aspect of SM without medical supervision, which is a potential risk of AMR. However, limited evidence is available related to LA in the community and the factors associated with them. This is the First study in Lebanon tackling this issue and shedding a light on the drastic sequences of this problem.

**Implications for policy and practice**

• Education programs aimed at promoting the rational use of antibiotics and emphasizing the importance of adhering to the prescribed regimen should be actively encouraged. These programs should target healthcare personnel, particularly physicians, as well as community members.
• Enforcing regulations on the dispensing of non-prescription antibiotics is imperative.
• Urgent action is needed to develop and implement national programs that promote the safe disposal of LA.
• In Lebanon, pharmacists should strictly adhere to the professional practice of not dispensing antibiotics without a valid medical prescription.

## 1 Introduction

Antimicrobial resistance (AMR) poses a significant and urgent threat to global public health [1,2]. Infection with antimicrobial-resistant organisms can be life-threatening and renders traditional treatment methods for common infectious diseases ineffective, leading to increased mortality rates, higher hospitalization rates, and greater financial burdens on healthcare systems [3]. The Centers for Disease Control and Prevention (CDC) reports that AMR contributes approximately 20 billion dollars in direct healthcare costs in the United States alone. Indirect costs of AMR are derived from present and future costs to society from the loss of outputs caused by a reduced labor supply and lower productivity attributable to increased morbidity and mortality.

Additionally, more than two million individuals in the United States are afflicted by antibiotic-resistant diseases each year, resulting in at least 23,000 deaths [4]. Furthermore, the emergence of multi-drug-resistant (MDR) gram-negative bacteria has further complicated the management of various infections, including pneumonia and urinary tract infections. The consequences of AMR include increased mortality rates, prolonged illness duration, higher healthcare costs, reduced treatment options, and the potential for widespread public health crises. AMR can exacerbate the burden of infectious diseases and pose significant challenges to healthcare systems worldwide, potentially leading to increased morbidity and mortality across various populations [5]. Multiple factors contribute to the rapid progression of AMR. One of the primary drivers is the improper and excessive use of antibiotics. A significant contributor to this issue is the widespread practice of SM, wherein individuals obtain antibiotics without proper medical prescriptions, and also utilize LA from previous treatments. This form of SM, particularly when it involves using antibiotics incorrectly or for inadequate durations, can have severe consequences and exacerbate the development of AMR [6]. Using LA from previous treatments to address a subsequent infection in the same patient presents a dual risk. Firstly, the pathogen causing the subsequent infection may have different susceptibility patterns, rendering the original antibiotic ineffective. Secondly, the efficacy of the LA may have been compromised due to improper or extended storage, exceeding expiration dates. This leads to suboptimal dosing, contributing to the development of AMR [7,8]. Furthermore, even when disposed inappropriately, such as being thrown in the sink or garbage, or flushed down the toilet, LA contributes to environmental pollution, exacerbating the issue [9]. Therefore, unlike many other drugs that, when misused, primarily pose a risk to the individual patient, improper usage of antibiotics amplifies the global risk of increased resistance dissemination and compromises the concept of One Health which emphasizes the need for cooperation among various disciplines, including human and veterinary medicine, environmental science, and public health, to address health issues such as AMR comprehensively. This approach acknowledges that the health of humans is closely linked to the health of animals and the environment, and aims to promote the well-being of all three in an integrated manner [10,11].

In recent years, there has been growing concern about LA. Studies have found that around 25% of Americans may have LA at home, and those who keep them are more likely to engage in SM. Similar findings were observed in a study across 19 European countries, highlighting the strong link between the presence LA at home and SM [12].

The situation in Lebanon regarding antibiotic consumption is particularly noteworthy. The country grapples with high levels of antibiotic misuse, as nearly half of the population engages in SM with antibiotics, and over 30% of antibiotics are dispensed without a prescription [[13], [14], [15]]. This has resulted in a significant increase in the overall community consumption of antibiotics, as measured by defined daily doses per 1000 inhabitants per day (DDD/1000 inhabitants/day), rising from 18.71 in 2004 to 31.26 in 2016 [16]. These findings can be attributed to several factors. These may include easy access to antibiotics without a prescription, limited enforcement of regulations regarding antibiotic sales, cultural norms and beliefs surrounding healthcare and self-medication, lack of awareness about the risks of antibiotic misuse, and challenges in accessing healthcare services. Additionally, factors such as the cost of healthcare, distrust in the healthcare system, and the influence of social networks and media may also contribute to the prevalence of SM with antibiotics and high antibiotic consumption in the Lebanese community.

Furthermore, surveillance of AMR in Lebanese hospitals has reported alarming findings. The prevalence rate of methicillin-resistant *Staphylococcus aureus* (MRSA) stands at 27.6%. Additionally, the production rates of extended-spectrum beta-lactamase in *Escherichia coli* and *Klebsiella* spp are 32.3% and 29.2%, respectively. *Acinetobacter* spp exhibits high resistance to most antimicrobials, while susceptibilities of *Pseudomonas* spp to piperacillin-tazobactam and imipenem are lower than 80%, indicating significant rates of AMR that necessitate urgent action [17]. Moreover, there is widespread availability of antibiotics in community pharmacies, sold without a medical prescription, often in the form of packed boxes rather than unit-dose pills. This practice encourages SM and contributes to the accumulation of LA [18].

However, there is a dearth of studies in Lebanon examining LA, including the various influences and associated factors. Therefore, the present national study aimed to assess the prevalence of LA in Lebanese households and investigate the factors contributing to their presence.

## 2 Methods

This is a cross-sectional regular phone survey, using the government data base of landlines, targeting Lebanese households with landline phone numbers spread across Lebanon between March and October 2022.

### 2.1 Definition of leftover antibiotics

“Leftover antibiotics”" refers to any unused or remaining antibiotics that were prescribed to a person for the treatment of a bacterial infection. Antibiotics are typically prescribed for a specific duration, and it's important for patients to complete the entire course of antibiotics even if their symptoms improve before the medication is finished [19].

### 2.2 Sample size determination

The sample size was determined using Epi-Info version 7.2, assuming an expected prevalence of LA of 50% and a margin of error of 5%. Thus, a minimum of 384 households was required to achieve a 95% confidence level. To account for potential incomplete interviews and multivariate analyses, a total of 600 households were ultimately targeted.

Systematic random sampling was initially employed to select landline phone numbers from a government list of Lebanese household phone numbers (Al Dalil Al Madani). The distribution of phone numbers in the sample was proportional to their distribution across Lebanese governorates to obtain a more representative sample. As a result, 176 households (35.22%) were selected from Mount Lebanon, 109 (21.75%) from the North, 96 (19.34%) from the South, 72 (14.36%) from Bekaa, and 47 (9.33%) from Beirut.

The selection of phone numbers followed a systematic random sampling approach, choosing every fifth number on the list. In cases where there was no response to the phone call twice in succession, another number was chosen.

### 2.3 Respondents’ selection

The individual responsible for managing medications at home, including purchasing, administering, storing, and discarding, was contacted and invited to participate in the survey via a phone call. If the respondent was not the person in charge of medication management, we requested to provide us with the contact information of the responsible party. We contacted the responsible person and questioned them about LA.

Our target was to interview the individual responsible for managing medications, particularly the person in charge of storing and discarding them. In cases where multiple individuals share medication-related responsibilities, one may handle the procurement while another oversees proper administration, storage, and disposal. We made sure to address this aspect during the respondent selection process.

### 2.4 Data collection

Data was collected through phone call surveys conducted by trained pharmacists. The surveys involved completing a pre-established questionnaire. Initially, the questionnaire was developed in English and then translated into Arabic. To ensure accuracy and validity, a back-translation into English was conducted, and a comparison between the two versions was made to identify any differences and ensure clarity. A pilot study was subsequently conducted, involving 10 randomly selected participants, to test the questionnaire over the phone. Based on feedback from these participants, minor modifications were made to the wording of certain questionnaire phrases.

The development of the questionnaire was based on a comprehensive literature review [7,20,21]. The first section of the questionnaire focuses on gathering data about the socio-demographic characteristics of the individual responsible for medication management at home. This includes information such as age, marital status, and education level. Other information collected includes the region of residence, number of family members, presence of children and elderly individuals in the household, presence of insurance, presence of a family member with a medical profession, presence of a family member who reads scientific information about drugs (including drug leaflets), and the crowding index. The crowding index measures the level of overcrowding in the household, with crowding considered to occur if there is more than one person per room. Severe crowding is defined as having more than 1.5 persons per room, excluding bathrooms, balconies, porches, foyers, hallways, and half-rooms [22].

The second part of the questionnaire focused on collecting relevant data related to LA. This included information about the availability of LA in the household, the specific names of the antibiotics, their dosage forms, and expiration dates. The questionnaire also inquired about the prescriber of the antibiotics, distinguishing between physicians, pharmacists, and SM (where antibiotics are obtained from a pharmacy without a prescription, without consulting a medical doctor). Additionally, participants were asked to provide information about the disease for which the antibiotics were taken and the reasons for having LA after their use.

### 2.5 Statistical analysis

Statistical analysis was conducted using SPSS version 26. Initially, a descriptive analysis was performed to assess the prevalence of LA in Lebanese households and provide an overview of the socio-demographic characteristics of the households and the individuals responsible for medication management at home. Subsequently, a bivariate analysis was conducted, using the presence of LA as the dependent variable, in order to test for any statistically significant relationships with the independent variables. The bivariate analysis employed methods such as Chi-Square and Fisher Exact tests for qualitative variables, with the former being a parametric test and the latter a non-parametric test. Additionally, the Student's t-test was utilized for analyzing relationships between quantitative dependent variables and qualitative independent variables.

For the logistic regression analysis, all independent variables (socio-demographic characteristics of the household and the individual responsible for medication management) with a p-value less than or equal to 0.2 from the bivariate analysis were included, and the dependent variable was having LA at home. The backward model was used to identify the factors associated with having LA. Collinearity between variables was assessed, the Hosmer-Lemeshow test was examined for insignificance, and the Omnibus test was assessed for significance in the logistic regression analysis. A p-value less than 0.05 was considered statistically significant.

## 3 Results

A total of 494 participants from various locations in Lebanon who fully completed the questionnaire were included in the study. However, to reach this sample size, 600 landline phone numbers were contacted, resulting in a response rate of 82%.

The primary cause of non-response to the interview was largely due to unavailability, stemming from factors such as travel or inactive contact numbers. Additionally, some respondents cited being occupied with domestic matters and a lack of time as reasons for non-participation.

### 3.1 Households’ characteristics in general

The majority of respondents were female (75.50%), and the mean age of participants was 46.58 ± 12.63 years. Regarding children, 15.59% of the contacted families reported having children under the age of 5, while approximately one-third (29.35%) had children aged between 5 and 12 years. The presence of elderly individuals in households accounted for only 30.77%. About three-quarters of the households stated that they had insurance coverage, and 41.09% reported having healthcare professionals among the family members. Additionally, half of the participants indicated that they had family members who read drug-related information. When considering the crowding index, almost half (49.19%) of the households were classified as having a high socioeconomic level. These results are summarized in Table 1.

**Table 1:** Socio-demographic characteristics of the participants.

Socio-Demographic Characteristics	Number (total = 494)	Percentage
**1. Participant Gender**		
Male (Father, son)	61	12.35
Female (Mother, daughter, aunt)	373	75.50
Non- specified	60	12.15
**2. Participant Marital Status**		
Married	370	74.89
Single	71	14.37
Widowed & divorced	53	10.72
**3. Level of Education**		
Less than University	232	46.96
University degree	262	53.04
**4. Age**		
Mean ± SD = 46.58 ± 12.63		
Minimum = 18		
Maximum = 80		
**5. Presence of Children Younger than 5 Years Old**		
0	417	84.41
1	56	11.34
+1	21	4.25
**6. Presence of Children 5 to 12 Years Old**		
0	349	70.65
1	80	16.19
+1	65	13.16
**7. Presence of Elderly**		
Yes	152	30.77
No	342	69.23
**8. Insurance**		
None	124	25.10
Private	60	12.15
Public	267	54.05
Public and private	43	8.7
**9. Presence of Healthcare Professionals in the Family**		
Yes	203	41.09
No	290	58.91
**10. Crowding Index**		
High >1	243	49.19
Moderate = 1	113	22.87
Low <1	138	27.94
**11. Reading Scientific Information Regarding Medications (Internet or Leaflet)**		
Yes	250	50.61
No	244	49.39
**12. Area of Residence**		
Beirut	46	9.31
Beqaa	70	14.17
Mount Lebanon	175	35.43
North	108	21.86
South	95	19.23

### 3.2 Households with LA

A total of 118 households reported having LA, indicating a prevalence rate of 23.89%. Among the different regions, the Mount Lebanon governorate had the highest proportion of households with LA (42.37%). The individuals responsible for managing medication in these households exhibited the following characteristics: the mean age was 43.45 ± 12.65 years, the majority were females (79.66%), 74.58% were married, and 55.93% held a university degree. Additionally, 19.49% of households with LA had children younger than 5 years old, 34.74% had children between 5 and 12 years old, and 36.44% had elderly members. Almost half of the households with LA were categorized as having a high socioeconomic level (44.92%), 51.06% had insurance coverage, and 55.08% did not have any household members employed in the medical field. Moreover, 62.71% of households claimed to have members who read drug-related information, including drug leaflets.

### 3.3 Characteristics of LA

**Fig. 1:**
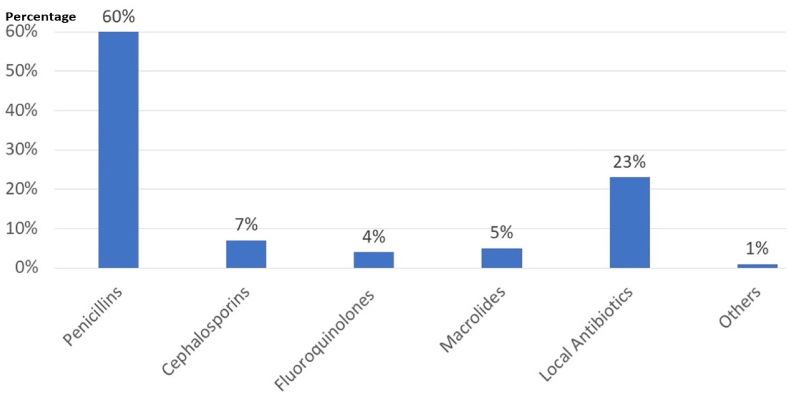
The frequency of leftover antibiotics in households sorted by their pharmacological class.

**Fig. 2:**
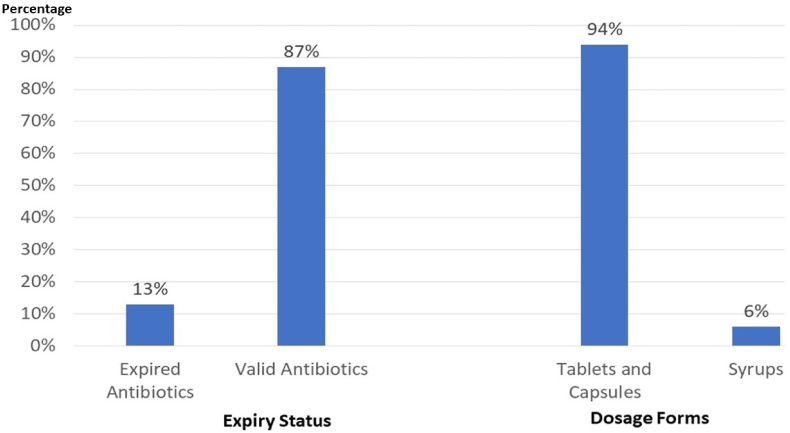
The expiry status and the dosage form of the leftover antibiotics in Lebanese households.

A total of 122 packs of LA were identified from the interview responses. Most of these packs (73,59.84%) belonged to the penicillin category, with 22.95% classified as intestinal antiseptics, and 0.82% falling into the “other" category. Among the 25 different antibiotics identified, amoxicillin + clavulanic acid was the most commonly found LA at home, accounting for 36.89% of the total. The results are demonstrated in Fig. 1. The majority of the antibiotics (94%) were in tablet or capsule form, while 6 % were in syrup form. Regarding expiration dates, it was determined that 106 antibiotics (86.89%) were still within their valid expiry dates, whereas 16 antibiotics (13.11%) had expired. The results are illustrated in Fig. 2.

Notably, the respondents indicated that their preferred methods for discarding expired antibiotics included disposing of them in the household trash or flushing them down the toilet.

Regarding the sources of LA, the majority were initially prescribed by physicians (61.02%), while approximately a quarter of them were dispensed by pharmacists without a medical prescription (22.03%).

In terms of the underlying diseases for which antibiotics were prescribed, respiratory infections accounted for almost half of the indications (47.46%), while gastrointestinal infections represented approximately a quarter of the cases (25.42%).

The study identified several factors contributing to the presence of LA in respondents' households. The main factor was the patient's clinical improvement after a short duration of antibiotic treatment, leading to the incomplete consumption of the prescribed course of therapy (48.31%). The second main factor was having remaining quantities of antibiotics after patients had completed the intended duration of treatment (33.90%). Other contributing factors included purchasing extra boxes of antibiotics (7.63%), experiencing side effects that resulted in discontinuation of the prescribed antibiotic course (5.08%), forgetting to continue the treatment for the intended duration (3.39%), and other miscellaneous reasons. The results are presented in Table 2.

**Table 2:** The characteristics of leftover antibiotics.

Leftover Antibiotics Characteristics		N (%) (out of 118)
**Antibiotics prescribed by**	Physician	72 (61.02)
	Pharmacist	26 (22.03)
	Auto-medication	20 (16.95)
**Reasons of prescription**	Respiratory infection	56 (47.46)
	Gastrointestinal infection	30 (25.42)
	Urinary infection	15 (12.71)
	Skin infection	14 (11.86)
	Other	3 (2.55)
**Reasons of leftover**	Felt Better	57 (48.31)
	The duration of treatment was over before the box was finished	40 (33.90)
	Bought too much quantity	9 (7.63)
	Side effects	6 (5.08)
	Forgot to continue	4 (3.39)
	Other	2 (1.69)

Furthermore, 42.37% of respondents indicated that they would consume LA without consulting a healthcare professional if they acquired the same illness again.

### 3.4 Bivariate analysis

The results of the bivariate analysis unveiled significant associations between the presence of LA and several factors. To begin, there was a notable association between the respondents' level of education and the presence of LA. University degree holders exhibited a higher prevalence of LA compared to those with lower educational degrees (p = 0.031). Furthermore, a significant association emerged between the presence of LA and the area of residence. Respondents residing in Mount Lebanon had a higher prevalence of LA (p = 0.044). Additionally, insured respondents demonstrated a higher prevalence of LA compared to those without medical insurance (p = 0.03).

Moreover, respondents who read scientific drug-related information displayed a higher prevalence of LA compared to those who did not (p = 0.006).

Finally, a significant association was observed between the age of the respondents and the presence of LA (p = 0.005), as illustrated in Table 3.

**Table 3:** Bivariate analysis of the factors associated with leftover antibiotics.

Independent Variable	Leftover Antibiotics No (total = 376) Number (%) Or Mean ± STD	Leftover Antibiotics Yes (total = 118) Number (%) Or Mean ± STD	p-value
**Region**			
Beirut	30 (7.98)	14 (11.86)	0.044*
Beqaa	50 (13.30)	20 (16.95)	
Mount Lebanon	126 (33.51)	50 (42.37)	
North	90 (23.94)	18 (15.25)	
South	80 (21.27)	16 (13.57)	
**Participant Gender**			
Female	280 (74.47)	94 (79.66)	0.021*
Male	43 (11.44)	19 (16.10)	
Non-specified	53 (14.09)	5 (4.24)	
**Age**	47.51 ± 12.50	43.45 ± 12.65	0.005*
**Marital Status**			
Single	51 (13.56)	23 (19.49)	0.05
Married	287 (76.33)	88 (74.58)	
Divorced or widow	38 (10.11)	7 (5.93)	
**Educational Level**			
Lower than university	209 (55.59)	52 (44.07)	0.031*
University	167 (44.41)	66 (55.93)	
**Insurance**			
None	101 (26.86)	22 (18.64)	0.03 *
Private	40 (10.64)	21 (17.80)	
Public	208 (55.32)	59 (50)	
Private & Public	27 (7.18)	16 (13.56)	
**Presence of children younger than 5 years old**			
0	322 (85.64)	95 (80.51)	0.353
1	39 (10.37)	18 (15.25)	
+1	15 (3.99)	5 (4.24)	
**Presence of children 5 to 12 years old**			
0	272 (72.34)	77 (65.25)	0.165
1	62 (16.49)	18 (15.25)	
+1	42 (11.17)	23 (19.50)	
**Presence of Elderly**			
Yes	101 (26.86)	75 (63.56)	0.005*
No	275 (73.14)	43 (36.44)	
**Presence of Healthcare Professionals in the Family**			
Yes	150 (39.89)	53 (44.92)	0.377
No	226 (60.11)	65 (55.08)	
**Crowding Index**			
High >1	190 (50.53)	53 (44.92)	0.564
Moderate = 1	85 (22.61)	28 (23.73)	
Low <1	101 (26.86)	37 (31.35)	
**Reading scientific information about medications (internet or drug leaflets)**			
Yes	177 (47.07)	74 (62.71)	0.006*
No	199 (52.93)	44 (37.29)	

*Indicates significant p-value <0.05.

### 3.5 Multivariate analysis

Following a logistic regression analysis conducted using the backward method, several significant associations emerged. Households with one elderly person had notably higher odds of having LA when compared to households without any elderly individuals (p = 0.002; OR = 2.23; 95% CI = 1.33–3.73). Additionally, residing in the Mount Lebanon area (p = 0.019; OR = 2.28; 95% CI = 1.14–4.56) and the age of the household member responsible for antibiotics (p- = 0.002; OR = 0.97 95% CI = 0.95–0.98) were also found to be significantly associated with the presence of LA, as detailed in Table 4.

**Table 4:** Logistic regression for the factors associated with leftover antibiotics.

Variable	Exp B]OR	CI 95%	p-value
**Region** Reference = South			
Beirut	2.07	0.85–5.01	0.107
Beqaa	2.07	0.92–4.63	0.078
Mount Lebanon	2.28	1.14–4.56	0.019
North	1.09	0.47–2.48	0.836
**Age**	0.97	0.95–0.98	0.002
**Elderly** Reference = No			
Yes	2.23	1.33–3.735	0.002

## 4 Discussion

This study revealed a prevalence of 23.89% for LA in Lebanese households. When compared to other countries, this prevalence varied, with some countries reporting lower rates and others reporting higher rates. For instance, a study in the United Kingdom found that only 6% of households had LA [23]. In Brazil, a higher prevalence of 34% for LA was observed, with a particularly strong association with amoxicillin + clavulanate at 42%, similar to our study's findings of 36.89% [24]. Furthermore, a global survey of LA in 11 countries discovered a prevalence of 51.9% among respondents who obtained antibiotics through new prescriptions or from medical professionals [25].

The primary factor associated with LA was the presence of an elderly member in the household. This could be explained by the fact that elderly individuals often have more frequent and complex healthcare needs, which may result in a higher likelihood of being prescribed antibiotics. Additionally, elderly individuals may have a higher incidence of chronic health conditions, leading to more prolonged or recurrent antibiotic use. Furthermore, older individuals may be more likely to retain medications, including antibiotics, due to factors such as forgetfulness, confusion about medication regimens, or a desire to keep medications on hand for future use. These factors collectively contribute to a higher likelihood of antibiotic leftovers in households where elderly individuals reside. A review of antimicrobial use in the elderly revealed that individuals aged ≥65 years used more antimicrobials, with a rate of 1.10 per person per year, compared to 0.88 antimicrobials used per person per year in individuals aged 0–64 years [26]. Age is a well-established risk factor for infection. Furthermore, several studies revealed increased non-compliance to antibiotics prescription and overuse by elderly [[26], [27], [28]].

While our study identified certain factors associated with LA, it is important to note that there are many others to consider. One example is the packaging of antibiotics, as countries that deliver them in packs have been found to have a higher prevalence of LA compared to those that use non-pack forms. For instance, China and Mexico, which both utilize a box delivery system, exhibited respective prevalence rates of 90% and 81% for LA. On the other hand, the Netherlands and the USA, which employ a non-box delivery system, had prevalence rates of 13.5% and 24.1%. Although Lebanon also employs a box delivery system, its prevalence of LA was not as high as seen in China and Mexico [7].

In our study, the attitude of “feeling better" was found to be the most common reason (48.31%) for discontinuing the antibiotic course and resulting in LA. A previous study reported that patients often stop taking antibiotics once they perceive an improvement in their symptoms [29]. Additionally, a large survey conducted in nine countries found that 10–47% of patients admitted to not completing a full course of antibiotic therapy, with 4–41% of them keeping the LA for future use [6]. In this context, it is worth mentioning that the duration of antibiotic treatment is a crucial aspect of managing bacterial infections, and it should be determined by a healthcare professional based on the site and severity of infection, the patient's overall health, and whether the infection is caused by MDR bacteria. Completing the full course of antibiotics helps ensure that all the bacteria causing the infection are eliminated, reducing the risk of recurrence and AMR ([30]) Therefore, it's important to educate patients to adhere to the prescribed course of antibiotics exactly as directed by the healthcare provider, even if they start feeling better before finishing the medication [31,32].

In our study, respiratory infections were identified as the primary reason for LA, constituting 47.46% of cases. This aligns with findings from other studies, such as one conducted in Lebanon, which reported that a majority of antibiotics were prescribed for respiratory tract infections (41%) [33]. Similarly, research in England revealed that respiratory tract infections represented 60% of all antibiotic prescriptions in general practice. Studies in Turkey and India also indicated that respiratory tract infections were the most common reasons for antibiotic prescriptions [34,35]. Additionally, a review highlighted that respiratory tract infections, particularly streptococcal throat/throat infections and influenza/colds, were the most prevalent conditions for which individuals had taken antibiotics [7,36].

In terms of antibiotic prescription, physicians were identified as the primary healthcare professionals responsible for prescribing them, followed by pharmacists, while some individuals practiced SM. These findings align with the study conducted by Kardas et al., which reported that 70.9% of antibiotics were obtained through doctor prescriptions, 16.5% from medical professionals, and 12% were SM [7]. Similarly, Marlière et al. discovered that the primary source of antibiotics was a medical prescription, followed by pharmacist recommendations, with SM being the least common approach [24]. It is important to note that over one-third of the participants reported SM with LA, reusing them for similar illnesses. This behavior highlights a lack of understanding regarding the responsible use of antibiotics and the associated risks of SM. When antibiotics are used without proper diagnosis, they may not effectively treat the infection and can contribute to AMR. This can make future infections more challenging to treat and potentially life-threatening. Additionally, SM with antibiotics can mask the symptoms of underlying conditions that require different treatments. This delays the proper diagnosis, worsens the underlying condition, and increases the likelihood of complications [14,37,38].

Finally, the study revealed that participants having expired antibiotics tend to dispose them in trash or flush them down the toilet which can lead to environmental hazards by contaminating water sources and harming aquatic life. This not only poses a risk to the environment but also has broader implications for 'One Health,' the interconnected relationship between human health, animal health, and the environment. Furthermore, improper disposal is a potential source of AMR development in the environment, which can subsequently affect human and animal health by diminishing the effectiveness of antibiotics when they are needed most [[39], [40], [41], [42], [43]]. The appropriate way to dispose of expired antibiotics is by following local guidelines or consulting a healthcare professional. Unfortunately, national guidelines for antibiotics disposal are not developed in Lebanon. Moreover, a national antimicrobial sterwardship program should be implemented to combat AMR and limit all the factors associated with its increase.

Our study's strengths are rooted in its substantial sample size and its coverage of various Lebanese governorates. Additionally, it is the first study to address LA in Lebanon. Our study also shed light on the prevalence of SM with antibiotics.

Lastly, it is important to consider the limitations of the current study. Data collection was conducted through phone calls, which opens the possibility of recall bias. Additionally, respondents may have under-reported the presence of LA in order to please the researcher. Furthermore, non-oral forms of antibiotics were not taken into account in this survey, which may have led to an underestimation of the prevalence of LA. It is also worth noting that the generalizability of the results is limited to households with landline phones. It is important to recognize that this study was a cross-sectional survey, and therefore any associations observed between factors and the findings should not be interpreted as causality relationships.

## 5 Conclusion

In conclusion, our study found that nearly a quarter of Lebanese households had LA. Associated factors included the presence of an elderly individual in the household, the age of the family member responsible for antibiotics, and the residency region. The presence of LA significantly increases the likelihood of SM in the general population, which in turn contributes to the growing problem of AMR.

It is pivotal to implement measures to limit irrational antibiotic use and to promote the safe disposal of LA. In this context, it is essential to raise population awareness regarding judicious antibiotic use which includes taking antibiotics exactly as prescribed by healthcare providers, adherence to the duration of the prescribed regimen, and proper disposal of any LA instead of saving them for future use or sharing them with others. Healthcare providers especially pharmacists have a paramount role in advocating the judicious use and safe disposal of antibiotics by patient education. However, this could not be ultimately achieved without implementing national regulations to combat antibiotic misuse such as restricting the dispensing of antibiotics in community pharmacies by medical prescription and developing national guidelines for the safe disposal of antibiotics such as the take-back approach.

In conclusion, promoting the rational use and responsible disposal of antibiotics is crucial for safeguarding the health of both humans and animals and ensuring the sustainability of our shared environment in the context of ONE Health.

## Ethical approval

The study protocol was approved by the Institutional Review Board (IRB) committee of the Lebanese International University (2020RC-065-LIUSOP). The participants provided their informed consent to participate in the study before the interview. Confidentiality of the participants' information was ensured, and their data were kept anonymous.

## Funding

There are no funding claims to be declared.

## Declaration of competing interest

The authors declare that they have no known competing financial interests or personal relationships that could have appeared to influence the work reported in this paper.
